# Neurexin-1-dependent circuit activity is required for the maintenance of photoreceptor subtype identity in *Drosophila*

**DOI:** 10.1186/s13041-023-01073-3

**Published:** 2024-01-02

**Authors:** Gabrielle Lim-Kian-Siang, Arianna R. Izawa-Ishiguro, Yong Rao

**Affiliations:** 1 McGill Centre for Research in Neuroscience, Montreal, Canada; 2 Department of Neurology and Neurosurgery, Montreal, Canada; 3 Integrated Program in Neuroscience, McGill University Health Centre, 1650 Cedar Avenue, Montreal, QC H3G 1A4 Canada; 4 Centre for Research in Neuroscience, McGill University Health Centre, Room L7-136, 1650 Cedar Avenue, Montreal, QC H3G 1A4 Canada

## Abstract

In the human and *Drosophila* color vision system, each photoreceptor neuron (cone cell in humans and R7/R8 photoreceptor cell in *Drosophila*) makes a stochastic decision to express a single photopigment of the same family with the exclusion of the others. While recent studies have begun to reveal the mechanisms that specify the generation of cone subtypes during development in mammals, nothing is known about how the mosaic of mutually exclusive cone subtypes is maintained in the mammalian retina. In *Drosophila*, recent work has led to the identification of several intrinsic factors that maintain the identity of R8 photoreceptor subtypes in adults. Whether and how extrinsic mechanisms are involved, however, remain unknown. In this study, we present evidence that supports that the *Drosophila* transsynaptic adhesion molecule Neurexin 1 (Dnrx-1) is required non-cell autonomously in R8p subtypes for the maintenance of R8y subtype identity. Silencing the activity of R8p subtypes caused a phenotype identical to that in *dnrx-1* mutants. These results support a novel role for Nrx-1-dependent circuit activity in mediating the communication between R8 photoreceptor subtypes for maintaining the subtype identity in the retina.

**Supplementary Information:**

The online version contains supplementary material available at 10.1186/s13041-023-01073-3.

## Introduction

The diversity of specific neuronal cell types in the brain allows the formation of complex neural networks for sophisticated and complex computational tasks. Over the last two decades, extensive studies have led to the identification of a large number of intrinsic and extrinsic factors that regulate the generation of diverse neuronal cell types during embryonic development (e.g. [1–4]). However, much less is known about the mechanisms by which the identity of specific neuronal cell types is maintained throughout the life of an animal [5].

The mosaic of mutually exclusive sensory neuronal subtypes in the human and *Drosophila* color vision system, offers excellent opportunities for understanding the mechanisms controlling the specification and maintenance of neuronal subtype identity. Cone cells in the human retina are responsible for color vision. Human cone subtypes are defined by their photopigments, including red- (L-opsin), green- (M-opsin) and blue-sensitive opsin (S-opsin). Each cone cell subtype expresses only one type of opsin with the exclusion of the other two [6, 7], which is important for accurately processing color information. Recent studies reveal important roles for thyroid hormone signaling in specifying cone subtypes in mice and humans during development [8–11]. The mechanisms by which the identity of cone subtypes is maintained in the adult retina, however, remain unknown. The *Drosophila* visual system, whose architecture shows important similarities to that of the mammalian visual system [12], is an excellent model to study evolutionarily conserved mechanisms controlling neuronal development and function.

The *Drosophila* compound eye is comprised of ~ 750 ommatidia, each containing eight different types of photoreceptor cells (R cells) and 14 accessory cells. In most cases, only a single type of photo-sensitive visual pigment Rhodopsin (Rh) is expressed in each R cell. R1-R6 photoreceptors express Rh1 that detects light in a broad spectrum, and are responsible for motion detection [13, 14]. R7 and R8 photoreceptors are responsible for color vision [15, 16], which can be further classified into either short-wavelength pale (p) or long-wavelength yellow (y) subtypes depending on the Rhodopsin they express. Rh3-expressing R7p and Rh4-expressing R7y detect light in the UV-spectrum [16]. Rh5-expressing R8p and Rh6-expressing R8y detect blue and green light, respectively [16]. Different classes of R cells project their axons into distinct layers in the optic ganglia; while R1-6 axons innervate the lamina, R7 and R8 axons pass through lamina into the deeper medulla and establish synaptic connections within the M6 and M3 sub-layers, respectively [17]. In the medulla, axonal arbors are segregated into ~ 750 regularly spaced columns, each of which includes axons from R7 and R8 photoreceptors and L1-L5 lamina neurons [12, 17].

The development of the *Drosophila* color vision system involves the specification of R7 and R8 subtypes. In each developing ommatidium, R7 precursor cell makes a stochastic decision to become either Rh3-expressing R7p or Rh4-expressing R7y. It is reported that the transcription factor Spineless (Ss) is a key player in specifying R7 subtype identity, as *ss* mutations caused all R7 cells to become Rh3-expressing R7p [18]. R7p or R7y then instructs R8 precursor cell within the same ommatidium to adopt a Rh5-expressing R8p or Rh6-expressing R8y subtype, respectively [19]. It is reported that the signals from R7p are TGF-β ligands, which activate the growth regulator Melted (Melt) and thus inhibit the Hippo pathway kinase Warts (Wts) in R8 precursor cell to specify R8p subtype [19]. And the activation of Wts by upstream regulators such as Merlin (Mer), Kibra (Kib), and Lethal (2) giant larvae (Lgl), specifies R8y subtype [20].

Recent studies reveal several intrinsic factors that maintain the identity of R8 subtypes. It is shown that Mer and Lgl are not only required for specifying R8y subtype during development, but are also required for the maintenance of R8y identity [20]. Interestingly, the maintenance of R8y identity is also dependent on R8y-specific expression of Rh6 that acts in a cell-autonomous manner to repress the expression of Rh5 [21]. In R8p subtypes, the expression of the neuronal transcription factor Erect wing (Ewg) is required for maintaining the R8p subtype identity [22]. Whether and how extrinsic mechanisms are involved in maintaining the identity of R8 subtypes, however, remain unknown.

The Neurexin family proteins are type-1 transmembrane proteins, and are well-known transsynaptic adhesion molecules [23]. In mammals, there are three Neurexin members, including α-, β, and γ-Neurexins [23]. Accumulated evidence supports that mammalian Neurexins function presynaptically to regulate synapse properties by binding to their postsynaptic ligands [24]. Several studies also show that Neurexins are expressed in the vertebrate retina [25–27]. The function of Neurexins in the vertebrate retina, however, remains unknown.

The *Drosophila* genome possesses a single *neurexin 1* (*dnrx-1*) gene that encodes α-Neurexin [28–30], and a *neurexin*-like gene (*dnrx IV*) [31]. Among them, only Dnrx-1 is a true Neurexin, as the domain structure of Dnrx IV is more related to members of the CASPR/paranodin family [30]. Dnrx-1 has been shown to be required for synaptic development and synapse properties in the *Drosophila* central nervous system as well as at the neuromuscular junctions [28, 29]. In the *Drosophila* retina, it is reported that Dnrx-1 is required in R1-R6 photoreceptor cells for retinoid transport [32].

In this study, we reveal a novel non-cell-autonomous role for Dnrx-1 in maintaining the identity of R8 photoreceptor subtypes. We also show that manipulation of R8p-dependent circuit activity affected the maintenance of R8 subtype identity. Together, these results support the requirements of Nrx-1-dependent circuit activity for the maintenance of R8 subtype identity.

## Results

### *nrx-1* mutations affected the identity of R8 photoreceptor subtypes

**Fig. 1:**
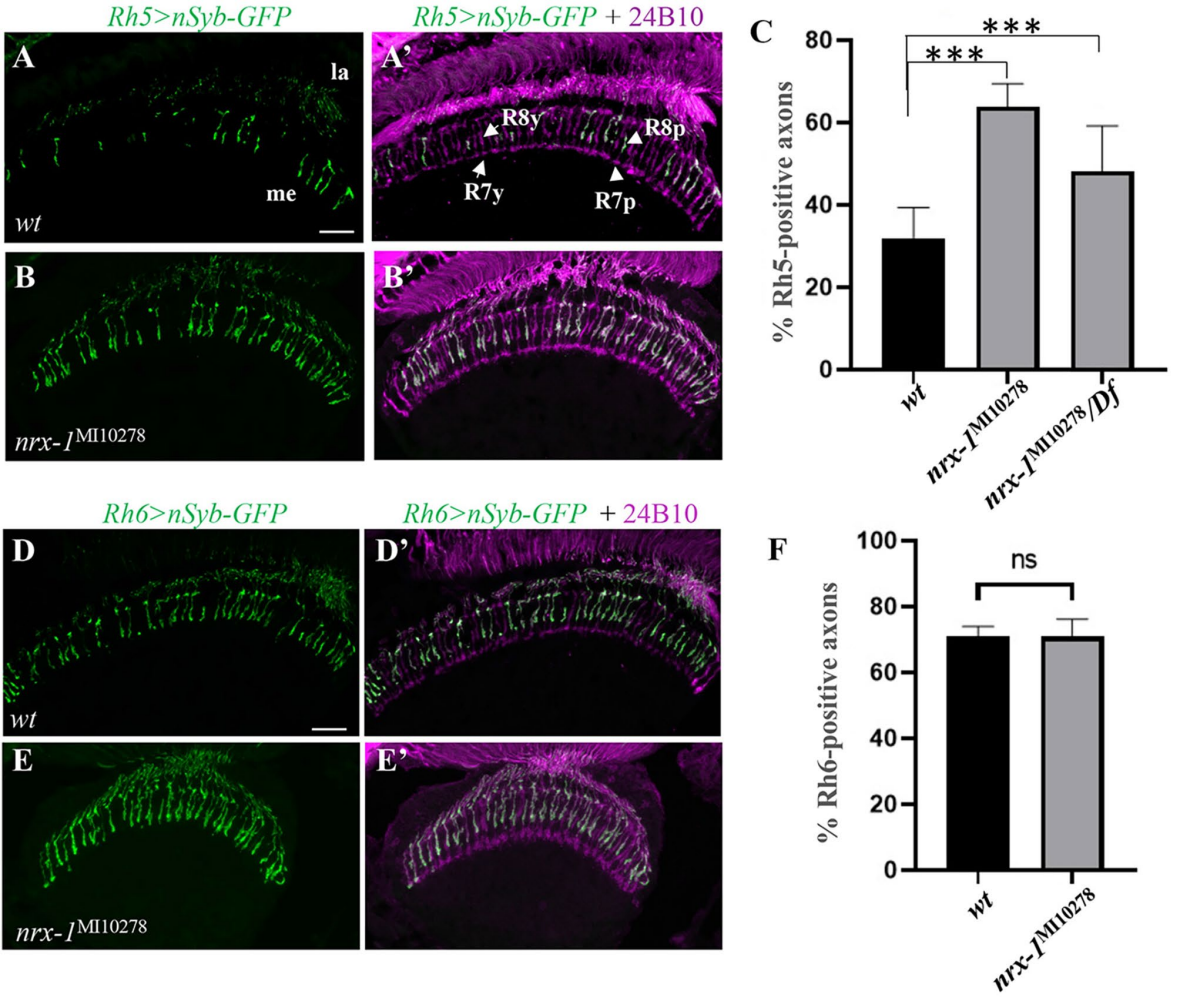
*dnrx-1* mutations disrupted Rh5 restriction in the retina. (**A**-**C**) Frozen sections of adult heads expressing *UAS-n-Synaptobrevin-GFP* (nSyb-GFP) under control of the R8p-specific driver *Rh5*-*GAL4* (i.e. *Rh5 > nSyb-GFP*), were stained with anti-GFP (green) and MAb24B10 (magenta). MAb24B10 recognizes the cell adhesion molecule Chaoptin expressed in all R-cell axons [54]. (**A** and **A**’) In wild type (n = 8), R1-R6 axons terminate in the lamina (la), while R7 and R8 axons pass through the lamina and terminate at distinctive layers of the medulla (me), M6 and M3 respectively. Among R8 axons, about 30% and ~ 70% were GFP-positive R8p axons (arrowhead) and GFP-negative R8y axons (arrow), respectively. (**B** and **B**’) In *nrx-1*^MI10278^ homozygous mutants (n = 12), the number of R8p axons showed a dramatic increase. (**C**) The percentage of Rh5-positive axons were quantified. Compared to that in wild type, the percentage of Rh5-positive axons were increased significantly in *nrx-1*^MI10278^ homozygous mutants (***p < 0.001). Similar increase in the percentage of Rh5-positive axons was also observed in *nrx-1*^MI10278^*/Df(3R)Exel6191* transheterozygotes (n = 12; ***p < 0.001). Total number of axons counted in wild-type flies: Rh5-positive axons, 447; all R8 axons, 1366, *nrx-1*^MI10278^ homozygous mutants: Rh5-positive axons, 1687; all R8 axons, 2639, and *nrx-1*^MI10278^*/Df(3R)Exel6191* transheterozygotes: Rh5-positive axons, 1135; all R8 axons, 2415. (**D**-**F**) Frozen sections of adult heads expressing *UAS-nSyb-GFP* under control of the R8y-specific driver *Rh6*-GAL4 (i.e. *Rh6 > nSyb-GFP*), were stained with anti-GFP (green) and MAb24B10 (magenta). (**D** and **D**’) In wild type (n = 7), about 70% of R8 axons in the medulla were GFP-positive R8y axons. (**E** and **E**’) *nrx-1*^MI10278^ homozygous mutants (n = 8). (**F**) The percentage of Rh6-positive axons were quantified. Compared to that in wild type, no significant difference in the percentage of Rh6-positive axons was observed in *nrx-1*^MI10278^ homozygous mutants. ns, *p* > 0.05. Error bars indicate SD. Scale bar, 20 μm

In the course of analyzing the role of Dnrx-1 in the fly visual system, we examined if mutations in the *dnrx-*1gene affect the development of R8 photoreceptors. In wild type, R8 precursors make binary fate decision that gives rises to R8 subtypes with mutually exclusive expression of Rh5 and Rh6. In wild-type flies, about 30% and 70% of R8 photoreceptors are Rh5-expressing R8p and Rh6-expressing R8y (Fig. 1A and A’ and 1 C), respectively, which are distributed stochastically throughout the retina [33].

In homozygous *nrx-1*^MI10278^ mutants, however, the percentage of R8 photoreceptor axons positive for the R8p-specific reporter (i.e. *Rh5 > GFP* [34]) increased significantly (Fig. 1B and B’ and 1C). The *nrx-1*^MI10278^ chromosome carries a MiMIC transposon inserted into the 8th intron in the *dnrx-1* gene [35]. The MiMIC transposon system contains a splice acceptor site followed by stop codons, and thus causes the truncation of the resulting Dnrx-1 protein in which the cytoplasmic domain, transmembrane region and ~ 50% of extracellular domain are deleted. Thus, *nrx-1*^MI10278^ is likely a strong loss-of-function allele, if not a null allele. Consistently, we found that transheterozygotes in which *nrx-1*^MI10278^ was placed over the *deficiency* chromosome *Df(3R)Exel6191* lacking the entire *dnrx-1* gene, displayed a phenotype that was similar to that in *nrx-1*^MI10278^ homozygous mutants (Fig. 1C).

The increase in the percentage of Rh5-positive axons in *dnrx-1* mutants may be due to a decrease in the number of Rh6-positive R8y subtypes. Alternatively, *dnrx-1* mutations may disrupt Rh5 restriction leading to the ectopic expression of Rh5 in R8y subtypes. To distinguish between these possibilities, we examined *dnrx-1* mutants by using a R8y-specific reporter (i.e. *Rh6 > GFP*). Compared to that in wild type (Fig. 1D and D’ and 1 F), however, no significant difference in the percentage of R8y axons was observed in *dnrx-1* mutants (Fig. 1E and E’ and 1 F). Together, these results suggest that Dnrx-1 is required for specification and/or maintenance of the R8y subtype identity by repressing the expression of Rh5 in R8y subtypes.

### R8p-specific knockdown of *nrx-1* disrupted the maintenance of R8y subtype identity by de-repressing Rh5 expression in R8y subtypes

**Fig. 2:**
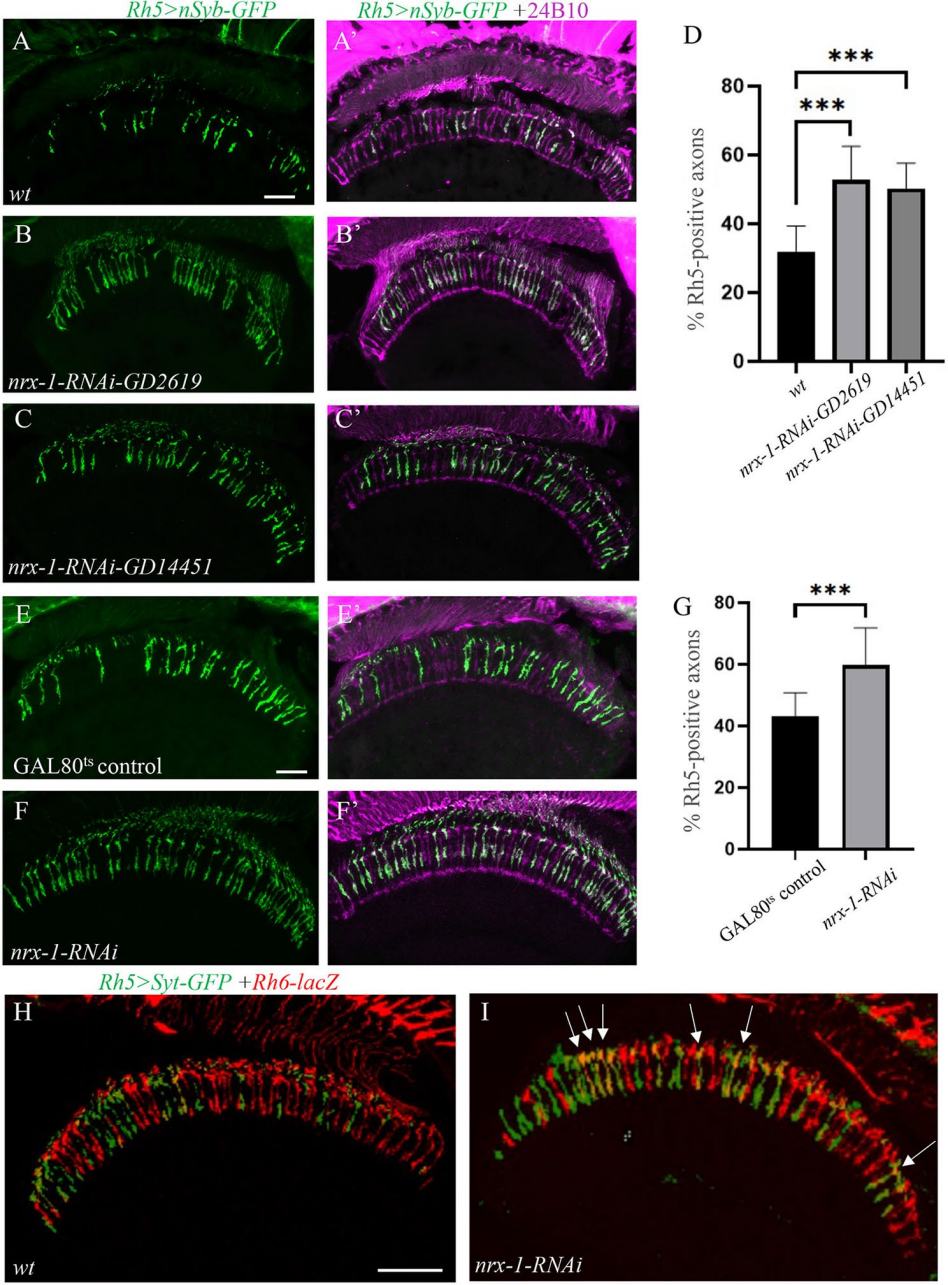
R8p-specific knockdown of *dnrx-1* disrupted the maintenance of R8y subtype identity by de-repressing Rh5 expression. (**A**-**D** and **E**-**G**) Frozen sections of adult heads expressing the Rh5 reporter *Rh5 > nSyb-GFP*, were stained with anti-GFP (green) and MAb24B10 (magenta). (**A** and **A**’) Wild type. (**B** and **B**’) Flies with R8p-specific expression of *UAS-nrx-1-RNAi-GD2619* (*n* = 13). (**C** and **C**’) Flies with R8p-specific expression of *UAS-nrx-1-RNAi-GD14451* (*n* = 14). (**D**) Significant increases in the percentage of Rh5-positive axons were observed with R8p-specific expression of two independent *dnrx-1*-RNAi transgenes *UAS-nrx-1-RNAi-GD2619* and *UAS-nrx-1-RNAi-GD14451*. ***p < 0.001. Error bars indicate SD. Scale bar, 20 μm. (**E** and **E**’) GAL80^ts^ control individuals (*n* = 13). (**F** and **F**’) Flies in which *dnrx-1* was specifically knocked down at adult stage (*n* = 12). (**G**) Knockdown of *dnrx-1* specifically at adult stage in R8p subtypes also significantly increased the percentage of Rh5-positive axons. ***p < 0.001. Error bars indicate SD. Scale bar, 20 μm. (**H** and **I**) Frozen sections of adult heads co-expressing Rh5 reporter *Rh5 > syt-GFP* and Rh6 reporter *Rh6-lacZ* were double-stained with anti-GFP (green) and anti-b-galactosidase (red). (**H** and **H**’) Wild type. (**I** and **I**’) In flies with R8p-specific expression of *nrx-1-RNAi-GD14451*, the Rh5 reporter was ectopically expressed in many R8y subtypes (arrows). Scale bar: 20 μm

To further determine the action of Dnrx-1, we performed cell-type-specific knockdown experiments using two independent *dnrx-1* RNAi transgenes (i.e. *UAS-nrx-1-RNAi-*GD2619 and *UAS-nrx-1-RNAi-GD14451*) targeting different regions in the *dnrx-1* gene. Interestingly, we found that the expression of *UAS-nrx-1-RNAi-*GD2619 or *UAS-nrx-1-RNAi-GD14451* under control of *Rh5*-GAL4 also caused a dramatic increase in the percentage of R8 axons positive for the R8p marker (Fig. 2B and B’, 2 C, 2 C’ and 2D), a phenotype identical to that observed in *dnrx-1* mutants (Fig. 1). Since *Rh5*-GAL4 was only expressed in R8p photoreceptors after the specification of R8p subtypes, this result indicates a role for Dnrx-1 in the maintenance of Rh5 restriction. Consistently, we found that knocking down *dnrx-1* specifically at adult stage also caused a similar phenotype in Rh5 restriction (Fig. 2F and F’ and 2G).

These results, together with that the percentage of R8 axons positive for the R8y marker *Rh6 > GFP* was not altered in *dnrx-1* mutants (Fig. 1E and E’ and 1 F), suggest that *dnrx-1* mutations disrupted the mutually exclusive expression of Rh5 and Rh6 in R8 subtypes by de-repressing the expression of Rh5 in Rh6-expressing R8y subtypes. Consistently, we found that R8p-specific *dnrx-1* knockdown disrupted the maintenance of Rh5 restriction leading to the formation of a new R8 subtype expressing both Rh5 and Rh6 (Fig. 2I).

Taken together, the above results suggest strongly that Dnrx-1 is required non-cell autonomously in R8p subtypes for the maintenance of R8y subtype identity by repressing Rh5 expression in R8y subtypes.

### Knockdown of *nrx-1* in R8 postsynaptic targets also affected the maintenance of Rh5 restriction

**Fig. 3:**
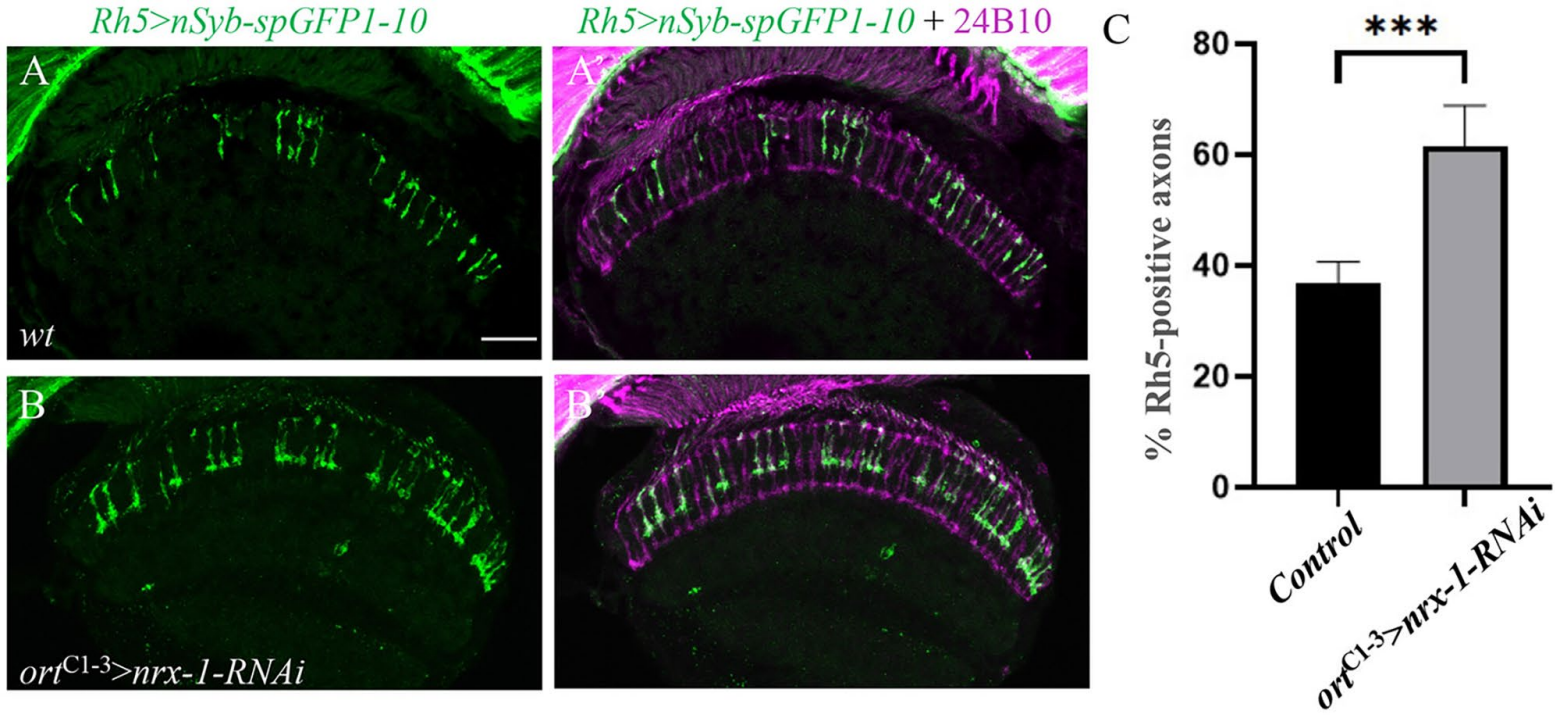
Knockdown of *dnrx-1* in R8 postsynaptic targets also affected the maintenance of Rh5 restriction. Frozen sections of adult heads expressing *lexAop-nSyb-spGFP1-10* under control of the Rh5-specific driver *Rh5-*lexA, were stained with anti-GFP (green) and MAb24B10 (magenta). (**A** and **A**’) Wild type (n = 10). (**B** and **B**’) Flies in which *dnrx-1* was specifically knocked down in R8 postsynaptic targets by placing *UAS-nrx-1-RNAi-GD2619* under control of the *ort*^C1–3^-GAL4 driver (n = 12). (**C**) The percentage of Rh5-positive axons were increased significantly when *dnrx-1* was specifically knocked down in R8 postsynaptic targets. ***p < 0.001. Error bars indicate SD. Scale bar, 20 μm

We then examined if knocking down *dnrx-1* in R8 post-synaptic target neurons affects the maintenance of Rh5 restriction. *UAS-nrx-1-RNAi-GD2619* was expressed in R8 postsynaptic target neurons in the medulla under control of the *ort*^C1–3^*-*GAL4 driver [36]. Interestingly, we found that like R8p-specific knockdown (Fig. 2), postsynaptic *dnrx-1* knockdown also disrupted the maintenance of Rh5 restriction as the percentage of R8 axons positive for the Rh5 reporter increased significantly (Fig. 3B and B’ and 3 C). This result suggests that Dnrx-1 is also required postsynaptically for the maintenance of Rh5 restriction in the retina.

### No sex-based effects were observed in the requirements of Dnrx-1 for the maintenance of Rh5 restriction

**Fig. 4:**
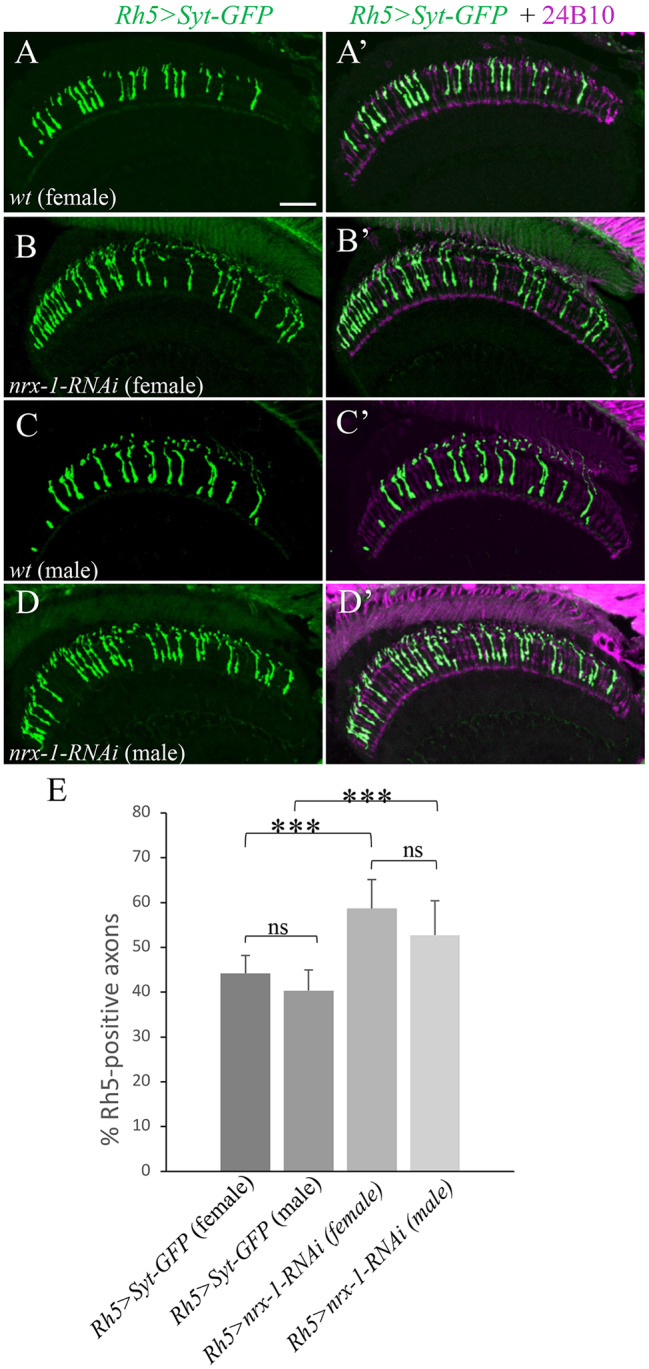
*dnrx-1* knockdown did not show sex-based effects on the maintenance of Rh5 restriction. Frozen sections of adult heads expressing the Rh5 reporter *Rh5 > Syt-GFP*, were stained with anti-GFP (green) and MAb24B10 (magenta). (**A**-**A**’) Wild-type female flies (n = 8). (**B**-**B**’) Female flies with R8p-specific expression of *nrx-1-GD14451* (n = 16). (**C**-**C**’) Wild-type male flies (n = 8). (**D**-**D**’) Male flies with R8p-specific expression of *nrx-1-GD14451* (n = 16). (**E**) No significant difference in the percentage of Rh5-positive axons between female and male *dnrx-1* knockdown flies was observed. ***p < 0.001. ns, *p* > 0.05. Error bars indicate SD. Scale bar: 20 μm

To determine the potential sex-based effects, we examined male and female *dnrx-1* knockdown flies separately. The results from male and female flies were compared. Compared to that in wild-type female (Fig. 4A and A’ and 4E) and male flies (Fig. 4C and C’ and 4E), both female (Fig. 4B and B’ and 4E) and male *dnrx-1* knockdown flies (Fig. 4D and D’ and 4E) displayed defects in the maintenance of Rh5 restriction, and no significant difference in the severity of the phenotype was observed between male and female knockdown flies (Fig. 4E).

### Silencing the activity of R8p subtypes caused a *nrx-1*-like phenotype

Neurexin family proteins are well known for their functions in the regulation of synaptic properties [24]. Our results revealing the requirements of Dnrx-1 in both R8p and their postsynaptic targets for Rh5 restriction and the maintenance of R8y subtype identity, suggest a role for R8p-dependent circuit activity in the maintenance of R8 subtype identity. To test this, we examined the effects of manipulating the activity of R8p subtypes on the maintenance of Rh5 restriction. Unlike mammalian photoreceptors in which light activation leads to hyperpolarization, light activation of *Drosophila* photoreceptors causes depolarization [37, 38], leading to the release of the neurotransmitter histamine that induces postsynaptic response in the optic lobe.

**Fig. 5:**
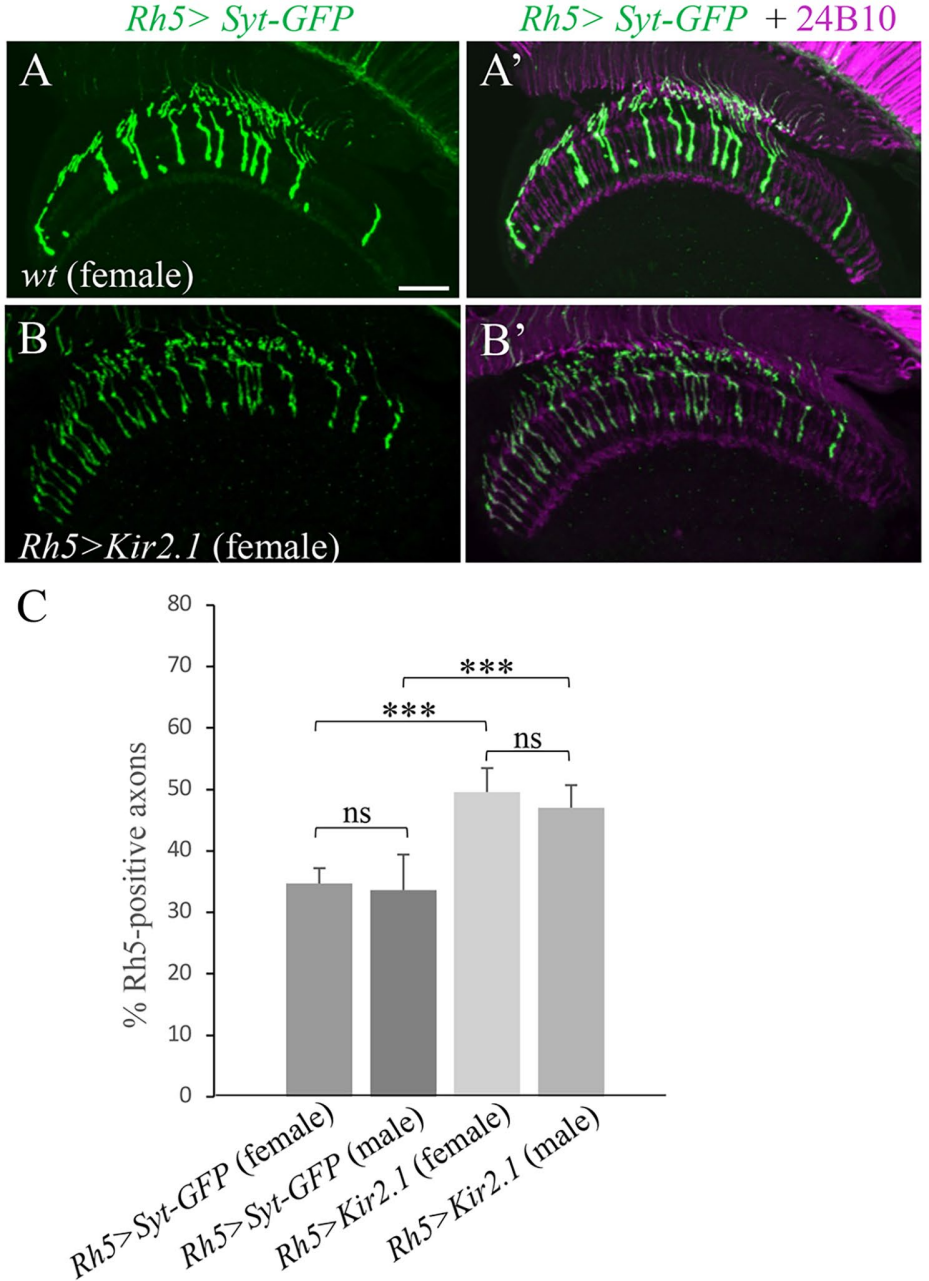
Silencing R8p caused a *dnrx-1*-like phenotype. Frozen sections of adult heads expressing the Rh5 reporter *Rh5 > Syt-GFP*, were stained with anti-GFP (green) and MAb24B10 (magenta). (**A**-**A**’) Wild-type female flies (n = 5). (**B**-**B**’) Female flies in which *Kir2.1* was specifically expressed in R8p subtypes (n = 5). (**C**) Silencing R8p with Kir2.1 significantly increased the percentage of Rh5-positive axons in both male and female flies. 5 male and 5 female flies in each genotype were examined. ***p < 0.001. ns, *p* > 0.05. Error bars indicate SD. Scale bar: 20 μm

To silence the activity of R8p subtypes, Kir2.1, an inwardly rectifying potassium channel, was specifically expressed in R8p subtypes by placing a *UAS-Kir2.1* transgene [39] under control of the *Rh5-*GAL4 driver. Kir2.1 decreases neuronal excitability by causing hyperpolarization [40]. Interestingly, we found that silencing the activity of R8p subtypes also caused a *dnrx-1*-like phenotype in the maintenance of Rh5 restriction in both male and female flies (Fig. 5B and B’ and 5 C). This result suggests strongly that R8p-dependent circuit activity is required for the maintenance of Rh5 restriction in the retina.

We then examined if increasing the activity of R8p subtypes rescues the *dnrx-1* phenotype. NaChBac [41], a bacterial voltage-gated sodium channel, was expressed specifically in R8p subtypes by placing a *UAS-NaChBac* transgene [42] under control of *Rh5-*GAL4. When expressed in *Drosophila* neurons, NaChBac could greatly increase the intrinsic excitability [42]. However, no rescue was observed in male or female *dnrx-1* knockdown flies (Supplementary Fig. 1C, 1C’ and 1D).

### Knockdown of *nrx-1* did not affect the formation of active zones in R8 axons

That Dnrx-1 is required both pre- and postsynaptically for the maintenance of Rh5 restriction, suggests a role for Dnrx-1 in R8 synaptic development and/or function, To assess this, we examined the expression of the active zone (AZ) protein Brunchpilot (Brp), the fly homolog of mammalian CAST/ELKS, which is an integral component of T-bar ribbons that tether presynaptic synaptic vesicles to the active zones and deliver them to the presynaptic membrane [43–46].

**Fig. 6:**
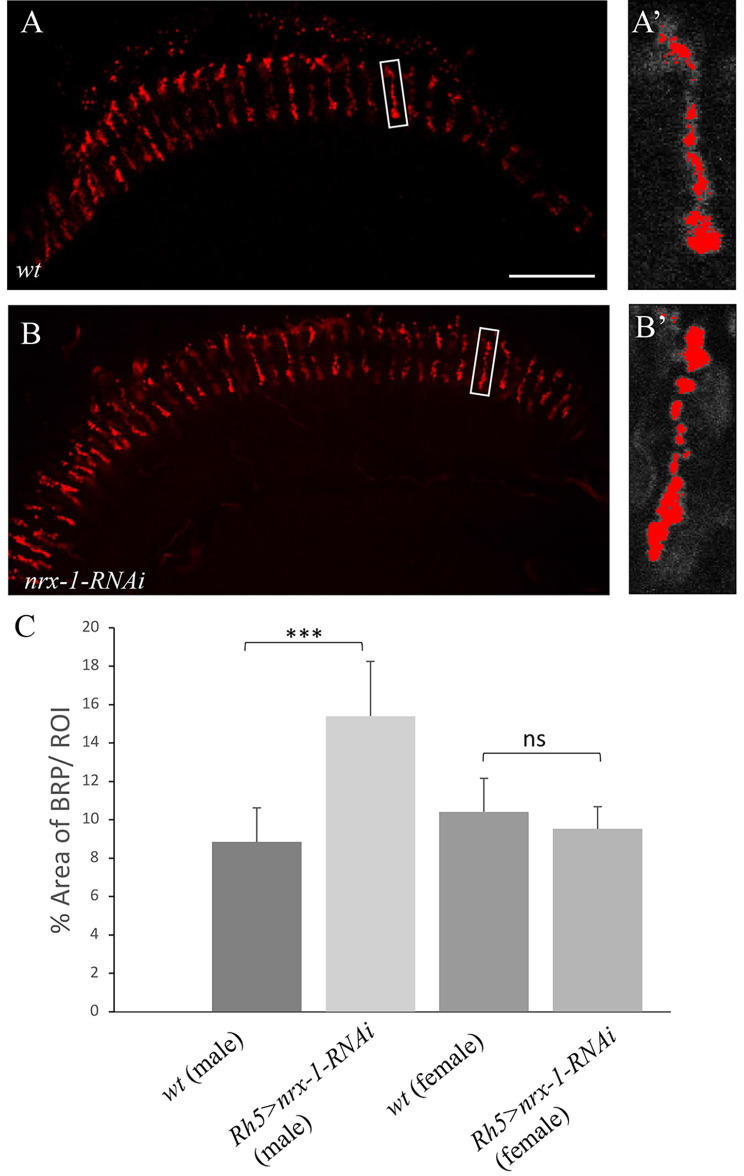
Knockdown of *dnrx-1* did not affect the formation of active zones in R8 presynaptic terminals. Frozen sections of adult heads expressing *Rh5/Rh6-brp-mCherry* were stained with anti-dsRed (Red). (**A**) Wild type. (**A**’) Enlargement of the boxed area in (**A**) (**B**) *dnrx-1* knockdown flies. (**B**’) Enlargement of the boxed area in (**B**). (**C**) The region of interest (ROI) was thresholded, and the percentage of Brp-positive area was quantified. While no decrease in the levels of Brp was observed in female *dnrx-1* knockdown flies (n = 8), there was an increase in the levels of Brp in male *dnrx-1* knockdown flies (n = 8). ***p < 0.05. ns, *p* > 0.05. Error bars indicate SD. Scale bar: 20 μm

Active zones in R8 axonal terminals were visualized with R8-specific expression of *Brp-mcherry*, which has been shown previously to faithfully label presynaptic structures in R8 photoreceptor axons [47]. Compared to that in wild type (Fig. 6A C), however, no decrease in the levels of Brp was observed in *dnrx-1* knockdown mutant axons (Fig. 6B C).

## Discussion

In this study, we provide several lines of evidence that support the importance of Dnrx-1-dependent R8p circuit activity for the maintenance of R8 photoreceptor subtype identity. Firstly, we show that Dnrx-1 is required non-cell autonomously in R8p subtypes for the maintenance of R8y identity by repressing the expression of Rh5 in R8y subtypes. Secondly, Dnrx-1 is also required postsynaptically for the maintenance of Rh5 restriction in the retina. And thirdly, like R8p-specific *dnrx-1* knockdown, silencing the R8p-dependent circuit activity disrupted the maintenance of Rh5 restriction. Together, these results reveal for the first time that activity-dependent communications between sensory neurons expressing distinctive sensory receptor genes play an important role in the maintenance of neuronal subtype identity.

The exact mechanism by which Dnrx-1 regulates R8p synaptic development and/or function remains unknown. In *Drosophila*, it is reported that Nrx-1 is required for synaptic development in the *Drosophila* central nervous system and at the neuromuscular junctions [28, 29]. However, no decrease in the levels of the AZ protein Brp was observed in *dnrx-1* mutant R8 axonal terminals (Fig. 6). This result raises at least two possibilities. For instance, Dnrx-1 may not be necessary for the recruitment of Brp into active zones, but instead required for the assembly of other AZ proteins for R8p presynaptic development. Alternatively, Dnrx-1 may be involved in the regulation of R8p synaptic function. In mammals, it is reported that neurexins are not required for synaptic development in presynaptic neurons of the calyx of Held in the mouse auditory system, but instead involved in coupling presynaptic calcium channels to neurotransmitter release sites at the calyx synapse [48]. Similarly, Dnrx-1 may couple Ca^2+^ influx to the release of the neurotransmitter histamine in R8p subtypes. This may explain why increasing the intrinsic excitability of R8p subtypes by expressing the bacterial voltage-gated sodium channel NaChBac did not rescue the phenotype in *dnrx-1* mutants (Supplementary Fig. 1), as the defects resulting from impaired coupling between Ca^2+^ influx and histamine release could not be ameliorated by an increase in R8p excitability.

That Dnrx-1 is required non-cell autonomously in R8p subtypes for repressing Rh5 expression in R8y subtypes (Fig. 2), together with the disruption of Rh5 restriction when silencing R8p subtypes (Fig. 5), suggest the existence of communications between R8p and R8y subtypes for the maintenance of Rh5 restriction and R8y identity. Since there is no direct contact between R8p and R8y subtypes, one likely possibility is that Dnrx-1 promotes the release of an unknown diffusible factor from R8p subtypes, which may act at a distance to repress the expression of Rh5 and thus maintain the identity of R8y subtypes. In mammals, it is reported that Neurexins are involved in neurotransmitter release in neurons [48] as well as the secretion of certain diffusible proteins in non-neuronal cells [49, 50]. Similarly, we speculate that Dnrx-1 promotes the release of an unknown diffusible factor from R8p subtypes in an activity-dependent manner for the maintenance of Rh5 restriction in the retina.

How does a R8p-derived diffusible signal repress the expression of Rh5 in R8y subtypes? Previous studies showing that continuous activation of the Hippo pathway in R8y subtypes is required for maintaining the R8y identity [20], raise the interesting possibility that the activity of the Hippo pathway in R8y subtypes is up-regulated by a R8p-derived diffusible signal for maintaining Rh5 restriction and R8y subtype identity. It is reported that TGF-β−family receptors such as Activin receptor Baboon (Babo) and the BMP receptor Thickveins (Tkv) are activated by their ligands to down-regulate the Hippo pathway for specifying R8p subtypes during development [19]. Thus, the R8p-derived signal may prevent the ectopic activation of the TGF-β pathway for maintaining the activity of the Hippo pathway in R8y subtypes at adult stage. Future studies are required to identify the R8p-derived signal, and elucidate the mechanisms by which the R8p-derived signal acts to repress the expression of Rh5 for the maintenance of R8y subtype identity.

Our results show Dnrx-1 is also required postsynaptically (Fig. 3). The exact action of Dnrx-1 in R8 postsynaptic neurons, however, remains unclear. In mouse hippocampal neurons, postsynaptic neurexins have been shown to modulate transsynaptic neurexin-neuroligin interactions via cis-interactions with neuroligin [51]. Similarly, Dnrx-1 may function on R8 postsynaptic neurons where it interacts in cis with neuroligin to modulate its transsynaptic interactions with Dnrx-1 on R8p subtypes. Such interactions may promote the release of certain diffusible factors from R8p subtypes for the maintenance of R8y subtype identity. Alternatively or additionally, Dnrx-1 may also mediate transsynaptic interactions between R8 postsynaptic neurons in the medulla and their target neurons in the lobula and lobula plate. The Dnrx-1-dependent circuit activity may also allow the release of certain diffusible factors from R8p postsynaptic target neurons, which may act in a distance to modulate R8y subtypes for Rh5 restriction. Future experiments can be performed to examine the effects of silencing the activity of R8 postsynaptic target neurons by expressing Kir2.1 on the maintenance of R8 subtype identity, which would provide further insights into the action of Dnrx-1 in R8 postsynaptic target neurons.

While our results provide strong evidence supporting the requirements of R8p activity for the maintenance of R8y subtype identity, it remains unknown whether the maintenance of R8p identity is also dependent on R8y activity. It would be of interest to examine if silencing the activity of R8y affects the maintenance of R8p identity. If the activity of R8y subtypes is indeed required for maintaining R8p identity, it is likely that it acts in a Dnrx-1-independent manner as our results show that *dnrx-1* mutations in R8y subtypes did not affect Rh6 restriction (Fig. 1). That the activity of one subtype affects the identity of a neighboring subtype could be indicative of a compensatory mechanism. The communications between different subtypes may allow the system to adjust the populations of R8 subtypes for optimizing color detection.

Similar mechanisms may also be used in other sensory systems for the maintenance of neuronal subtype identity. For instance, in the fly and vertebrate olfactory system, olfactory receptor neurons (ORNs) typically express a single olfactory receptor (OR) gene [52]. Olfactory receptor expression defines ORN classes, akin to how Rhodospin expression defines photoreceptor subtypes. Like that in the visual system, several intrinsic factors for the maintenance of OSN subtype identity have been identified [53]. Given the similarities between sensory neurons of the visual and olfactory system, it is likely that the communications between OSN subtypes are also important for the maintenance of their identity. It would be of interest to examine whether silencing the activity of one type of ORN alters OR expression in the surrounding ORNs.

## Conclusion

The present study presents evidence that suggests a novel role for the evolutionarily conserved transsynaptic adhesion molecule Dnrx-1 in the maintenance of R8 photoreceptors subtype identity. That Dnrx-1 is required both pre- and postsynaptically, together with the requirements of R8p activation, support the involvement of Dnrx-1-dependent R8p circuit activity. Dnrx-1 may promote the release of an unknown diffusible factor from R8p subtypes in an activity-dependent manner, which mediates the communication between R8p and R8y subtypes to maintain the R8y subtype identity. Given the evolutionary conservation of Neurexin family proteins, similar mechanisms may also be used in mammals to maintain neuronal subtype identity in adults.

## Materials and methods

### Genetics

Fly stocks are reared at 25 °C with 50% humidity and 12/12 h light–dark cycle. Following stocks were obtained from Bloomington *Drosophila* Stock Center (BDSC): *Rh5-*GAL4 (BDSC #7458), *Rh6-*GAL4 (BDSC #7459), *UAS-nSyb-GFP* (BDSC #6921), *UAS-Syt-GFP* (BDSC #6926), *nrx-1*^MI10278^ (BDSC #55,467), *and Df(3R)Exel6191* (BDSC #7670), *lexAop-nSyb-spGFP1-10* (BDSC #64,315), *tubP-*GAL80^ts^ (BDSC #7017), *Rh6-lacZ* (BDSC #8117), *UAS-NaChBac* (BDSC #9466), *Rh5/Rh6-brp-mcherry* (*BDSC#57,322*). Flies for *dnrx-1-RNAi* knockdown experiments were obtained from the Vienna *Drosophila* Resource center (VDRC): *UAS-nrx-1- RNAi-GD14451 (VDRC #36,326)* and *UAS-nrx-1-RNAi-GD2619 (VDRC #4306). UAS-Kir2.1* flies were provided by C. Lee at NIH. *Rh5-lexA* flies were provided by C. Desplan at New York University.

The *UAS*-GAL4 system was used for specific expression or knockdown of genes in flies. Genetic crosses were performed to generate following R8p and R8y control flies: *Rh5-*GAL4 *+ UAS-nSyb-GFP* (or *Rh5 > nSyb-GFP*), *Rh6-*GAL4 + *UAS-nSyb-GFP* (or *Rh6 > nSyb-GFP*), *or Rh5-*GAL4 *+ UAS-Syt-GFP* (or *Rh5 > Syt-GFP*). For phenotypic analysis of *dnrx-1* mutants, genetic crosses were performed to generate following flies: *Rh5 > nSyb-GFP; nrx-1*^MI10278^*/nrx-1*^MI10278^, *Rh5 > nSyb-GFP; nrx-1*^MI10278^*/Df(3R)Exel6191, and Rh6 > nSyb-GFP; nrx-1*^MI10278^*/nrx-1*^MI10278^. For R8p-specific *dnrx-1* knockdown, genetic crosses were performed to generate following flies: *Rh5 > nSyb-GFP + UAS-nrx-1-RNAi-GD2619/+, Rh5 > nSyb-GFP + UAS-nrx-1-RNAi-GD14451/+*, and *Rh5 > Syt-GFP + UAS-nrx-1-RNAi-GD14451/+.* For knockdown of *dnrx-1* in R8 postsynaptic targets, the LexA/LexAop system was used to label R8p axons in following flies: *ort*^C1–3^*-*GAL4/+; *Rh5-lexA > lexAop-nSyb-spGFP1-10 and ort*^C1–3^*-*GAL4 > *UAS-nrx-1-RNAi-GD2619/+; Rh5-lexA > lexAop-nSyb-spGFP1-10*.

For knocking down *dnrx-1* specifically at adult stage, genetic crosses were performed to generate following flies: *Rh5* > *nSyb-GFP; tubP-*GAL80^ts^/+ and *Rh5* > *nSyb-GFP + UAS-nrx-1-RNAi-GD2619/+; tubP-*GAL80^ts^/+. The flies were reared at permissive temperature (i.e. ≤25 ^o^C) prior to eclosion, which prevented the knockdown of *dnrx-1* by allowing the inhibition of GAL4 by GAL80. Immediately after eclosion, flies were reared at restrictive temperature (i.e. 30.5 ^o^C) for 5 days, which inactivated GAL80 and thus allowed *Rh5*-GAL4 to drive the expression of *UAS-nrx-1-RNAi-GD2619* in R8p subtypes in adult flies.

To examine if *dnrx-1* knockdown causes ectopic expression of Rh5 in R8y subtypes, genetic crosses were performed to generate following flies: *Rh5 > Syt-GFP/Rh6-lacZ* and *Rh5 > Syt-GFP + UAS-nrx-1-RNAi-GD14451/Rh6-lacZ*. To silence R8p subtypes, genetic crosses were performed to generate following flies: *Rh5 > Syt-GFP + UAS-Kir2.1*. To increase the excitability of R8p subtypes in wild-type or *dnrx-1* knockdown flies, genetic crosses were performed to generate following flies: *Rh5 > Syt-GFP + UAS-NaChBac* and *Rh5 > Syt-GFP + UAS-NaChBac + UAS-nrx-1-RNAi-GD14451/+*. To examine the effects of *dnrx-1* knockdown on the formation of active zones, genetic crosses were performed to generate flies with following genotypes: *Rh5-*GAL4*/+; Rh5/6-brp-mcherry/+* and *Rh5-*GAL4*/+, UAS-nrx-1-RNAi-GD14451/Rh5/6-brp-mcherry*.

### Cryostat

Adult fly heads were dissected 3 days post-eclosion (or 5 days post-eclosion for experiments involving the use of GAL80^ts^), and were fixed for 3 h on ice in 3.2% paraformaldehyde in phosphate buffer (PB, pH 7.2). The heads were then washed 3X using PB and were incubated overnight at 4 °C in 25% sucrose in PB. The following day heads were mounted in O.C.T. (Optimal Cutting Temperature) Compound, and subsequently cryostat sectioned at 10 μm thickness and placed onto Superfrost Plus slides (Fisher Scientific) using a Leica CM3050 S. Sections on slides were then blocked with 10% normal goat serum (NGS) in 0.5% PBT (phosphate buffer with Triton X-100) for 1 h, and then incubated with primary antibodies (MAb24B10 at 1:100 [Developmental Studies Hybridoma Bank or DSHB]; rabbit polyclonal anti-GFP at 1:1000 [Invitrogen], or mouse anti-b galactosidase [Sigma-Aldrich] at 1:100) overnight at 4 °C. The sections were washed 3X with 0.5% PBT, followed by incubation with secondary antibodies (anti-mouse AlexaFluor 647 at 1:750 or anti-rabbit AlexaFluor 488 at 1:750 [Invitrogen]) for 55 min at room temperature. The slides were then washed 3X with 0.5% PBT, and overlayed with 60 µL SlowFade Gold anti-fade (Invitrogen) per slide. Slides were each mounted with a glass coverslip, sealed with clear nail polish, and kept at 4 ^o^C until confocal microscopy (Olympus, Fluoview FV1000 LSM). Z-stack images were taken at 40x magnification.

### Whole-mount staining of adult heads

Adult heads were dissected in 0.3% PBT on ice 3 days post-eclosion. The dissected brains were fixed in 4% PFA for 20 min at room temperature on a nutator. Following fixation, the tracheas were removed to prevent the tissues from floating. PFA was then removed with a P-200 pipette and replaced with 0.5 ml 0.3% PBT. The brains were washed 3X in PBT by inverting the tube once and changing the PBT. After 3 short washes, the brains underwent 3 long washes: 0.5 ml PBT was added to the tube, which was placed on the nutator for 20 min at room temperature. After the washes, PBT was replaced with 0.5 ml 5% NGS in 0.3% PBT, and the tube was placed on the nutator for 30 min at room temperature. The brains were then incubated with primary antibodies (MAb24B10 at 1:100 or rabbit polyclonal ds-red [Sigma-Aldrich] at 1:500) for 2 nights at 4 °C. ds-red recognises Brp-mcherry expressed in R8 axons. The brains underwent 2 more short washes and 3 long washes with 0.3% PBT and were incubated with secondary antibodies (anti-mouse AlexaFluor 647 at 1:500 or anti-rabbit AlexaFluor 488 at 1:500 [Invitrogen]) for 2 h at room temperature. The brains underwent 2 more short washes and 3 long washes with 0.3% PBT. 200 µL SlowFade Gold anti-fade (Invitrogen) was then added to each tube. The brains were mounted on glass slides with a P-200 pipette. The brains were placed between the bridges to prevent tissue damage, and excess Slowfade was removed with a Kimwipe. A coverslip was placed over the bridges. The samples were sealed with clear nail polish and stored at 4 °C in a dark holder until confocal microscopy.

### Quantification of active zones in R8 axonal terminals

Confocal Z-stacks were taken at 60x magnitude and a step size of 0.5 μm, which was previously shown to be the ideal step size to prevent redundant quantification of presynaptic puncta [47]. The stacks were analyzed with ImageJ, and the percentage of area expressing Brp over a selected region of interest (ROI) containing one R8p axon was quantified.

### Statistical analysis

The number of Rh5- and Rh6-positive axons as well as all R8 axons were counted manually, and the percentage of R8 axons that were positive for Rh5 or Rh6 in each individual were quantified. Shapiro -Wilkes tests were used to determine if the datasets were best analyzed with parametric or non-parametric tests. Levene’s tests were used to determine variance homogeneity. Homoscedastic datasets were analysed using either the unpaired t-test (2 samples) or the one-way between-subject analysis of variance (ANOVA) test (≥ 3 samples), followed by post-hoc Dunnett’s testing. Heteroscedastic data was analysed using either a Welch’s t-test (2 samples) or a Kruskall-Wallis test (≥ 3 samples). When the resulting *p* value < 0.05, the statistical differences were considered significant.

### Electronic supplementary material

Below is the link to the electronic supplementary material.

Supplementary Material 1

## Data Availability

The datasets supporting the conclusion of this study are included in this article.
