# Supplementary material for: Neurexin-1-dependent circuit activity is required for the maintenance of photoreceptor subtype identity in Drosophila
Source: Mol Brain. 2024 Jan 2;17:2. doi: 10.1186/s13041-023-01073-3 (PMC10759516; doi:10.1186/s13041-023-01073-3)
Supplement: Supplementary file 1 — Supplementary Material 1 [file 13041_2023_1073_MOESM1_ESM.docx]

**Supplementary Material**

**

**

**Supplementary Figure 1**. Increasing R8p excitability did not rescue the phenotype in *dnrx-1* mutants. Frozen sections of adult heads expressing the Rh5 reporter *Rh5>Syt-GFP*, were stained with anti-GFP (green) and MAb24B10 (magenta). (A-A’) Wild type (n=10, 5 females and 5 males). (B-B’) Flies in which *UAS-nrx-1-RNAi-GD14451* was specifically expressed in R8p subtypes (n=10). (C-C’) *dnrx-1* knockdown flies in which *UAS-NaChBac* was specifically expressed in R8p subtypes (n=10, 5 females and 5 males). (D) Flies expressing *UAS-NaChBac* in R8p subtypes still showed an increase in the percentage of Rh5-positive axons, which were not significantly different from that in *dnrx-1* knockdown flies. ns, *p* > 0.05. Error bars indicate SD. Scale bar: 20 μm.
